# Crisis management for Patient Safety Officers: lessons learned from the Covid-19 pandemic

**DOI:** 10.1186/s13584-023-00577-6

**Published:** 2023-09-01

**Authors:** Ilya Kagan, Dana Arad, Riki Aharoni, Yossi Tal, Yaron Niv

**Affiliations:** 1 Nursing Department, School of Health Professions, Ashkelon Academic College, Ben Tzvi 12, 78211 Ashkelon, Israel; 2 Patient Safety Division, Ministry of Health, Jerusalem, Israel; 3 Risk Management and Patient Safety Advising Services, Raanana, Israel; 4 Adelson Faculty of Medicine, Ariel University, Ariel, Israel

**Keywords:** Patient Safety Officer, Risk management, Patient safety, Pandemic, Covid-19, Uncertainty, Initiative

## Abstract

**Background:**

There is no consensus for the role definition for Patient Safety Officers (PSOs) in healthcare during pandemics or other crises as opposed to their routine activities. This study aimed to examine the contribution of personality traits and systemic factors on the performance of PSOs during the pandemic, and to compare these variables during the first and third waves of the Covid-19 pandemic in Israel.

**Methods:**

This cross-sectional study invited 117 PSOs to complete a questionnaire addressing their role during the Covid-19 pandemic. The questionnaire included items concerning: Personal and socio-demographic characteristics; Uncertainty; Personal initiative; Burnout; Professional functioning**;** Patient Safety and Risk Management policies and practices; Organizational functioning; and Personal Involvement in risk management activities. Qualitative data was collected by two open-ended questions.

**Results:**

A total of 78 PSOs (67%) completed the questionnaire. The results revealed that many PSOs reduced their involvement in risk management processes or even left their position temporarily in order to return to their primary specialization as clinicians. Only 51.3% and 57.7% reported practicing risk management in the first and third waves, respectively. The three main factors that kept PSOs functioning were managerial support, mobilization of their team, and the belief in the importance of their position.

**Conclusions:**

A crisis generates uncertainty, a plethora of frequent and urgent tasks, and the need to adapt policy to changing circumstances and to the increased risks. The risk manager must be a member of the crisis management team and participate in every important discussion in order to represent essential staff and patient safety issues and ensure that these are fully addressed already in the early stages of planning.

## Background

When the Covid-19 pandemic started in Israel in February 2020, the Israeli Healthcare system entered emergency mode, with all resources directed towards providing healthcare for Covid-19 patients. The pandemic progressed in waves with the first wave between February and June 2020, the second wave between August and October 2020, and the third wave between December 2020 and February 2021 [1].

Under routine conditions, patient safety is expected to be an essential component of the healthcare system. The first and central axiom with respect to potential iatrogenic conditions is the Hippocratic Oath of “*primum non nocere*” (first, do no harm) [2]. The role definition of a risk manager or patient safety officer (PSO) in a medical institute involves the commitment to promote patient safety, prevent errors, and protect patients from harm by assessing potential risks and planning prevention as a safety net [3, 4]. In Israel healthcare system, the job responsibilities of PSOs are defined in circulars from the Director General of the Ministry of Health that address the organizational structure of the Patient Safety Units in hospitals [5] and in community health services [6]. PSOs are in charge of conducting the following activities: a) reactive—investigation of adverse events in order to draw conclusions and prevent them in the future; b) interactive activities—immediately following or during the occurrence of adverse events, aimed at minimizing the damages to the patient, the caregivers, and the organization; and c) proactive—aimed to recognize and minimize potential risks, and prevent latent failures [5, 6]. The majority of the time, the Patient Safety unit only engages in reactive and interactive activities.

During the pandemic, most efforts were directed to immediate care, testing, and vaccination. An estimated 10–15% of Covid-19 patients were hospitalized in an acute condition, and many required respiratory support. Age and chronic disease increased the risk for severe mortality and morbidity [7]. Challenges in providing rapid medical care and a shortage of hospital beds lead to changes in the type of care given, increased the potential occurrence of medical errors such as diagnostic errors [8], and decreased patients’ treatment quality and safety [9, 10]. Countries such as Italy, the US, and England reported a decrease in safety processes including safety rounds and quality control activities [10].

The role and responsibilities of the PSO (Patient Safety Officer) in a medical organization in crisis are rarely defined and are usually described in general terms. We were not able to find a comprehensive description of the role and responsibilities of a PSO in a pandemic or other crisis. In order to establish a benchmark, we approached other high-risk industries like aviation. Although we are aware of the many differences between healthcare and aviation, and appreciate that these may limit a direct transfer, it may be significant to mention that aviation served as a reference in establishing safety practices in healthcare, at least in the initial stages [11]. In this context, chapter 6 of the "Operator's Flight Safety Handbook" [12], deals with emergency responses and crisis management and details the planning, preparation, and action in a crisis. One of the recommendations refers to the appointment of a safety officer as a member of a CMC (Crisis Management Centre) that is established in a crisis situation. The safety officer may be tasked with planning the company’s emergency response and crisis management procedures, although in a large organization this may be the responsibility of a dedicated Emergency Planning department. Thus, it can be concluded that the aviation Safety Officer plays a crucial role in all stages of crisis management.

To the best of our knowledge, there is no role definition for a patient safety officer or risk manager in healthcare during a pandemic as opposed to their routine activities. Therefore, there is a need to explore the effect of personal and organizational aspects on the functioning and well-being of PSOs during the pandemic and to compare these aspects during the first and third waves of Covid-19.

### Aim

This study was designed: (a) to examine the contribution of personality traits and characteristics (uncertainty, initiative, and burnout) and systemic factors (organizational functioning) on the performance of PSOs, and (b) to compare these variables in the first and third waves of the Covid-19 pandemic in Israel. The study's findings will help in better identification of needs, more accurate job definitions and improved support for PSOs in future emergencies.

## Methods

### Sample

This cross-sectional study invited senior organizational PSOs from all the medical organizations in Israel, from hospitals and community services to participate. PSOs who were not active in their organization during this period for reasons of maternity leave, prolonged sick leave, or being abroad, were excluded from the study. The sample size was calculated using G-Power 3.1 software, which indicated that 54 subjects could provide a power of 0.95 with an effect size of 0.5 at a significance level of 0.05 for performing paired *t*-tests. The sample was increased by 15% to ensure the ability to perform advanced statistical analyses and to reduce problems of missing data due to compliance issues.

### Tools

Data were collected using a self-administered questionnaire that examined: (a) uncertainty; (b) initiative; (c) burnout; (d) patient safety and risk management activities; (e) PSO functioning; (f) organizational functioning; (g) personal involvement in risk management activities; and (h) socio-demographic characteristics. The questionnaire was based on existing tools that were modified for the purpose and the study population although the authors generated the "d" and "f" sections. Five experts in Quality and Patient Safety validated the final version of the tool. The experts were asked to check whether the questions were consistent with the study objectives and any problematic wording was corrected. In section "d", only the items for which there was full consensus were included. The responders were asked to consider the study variables as manifested in the first and third waves of the Covid-19 pandemic.

*Personal and socio-demographic characteristics* included gender, age, profession, employment (partial or full-time), professional seniority, and seniority in patient safety and risk management in the organization.

*Uncertainty* was measured using a 7-item tool that was constructed by Kagan, et al. [13], shortened by Melnikov et al. [14], and adapted to the Covid-19 pandemic [15]. The tool assesses uncertainty related to significant organizational events and changes. Items #3 and 7 were recoded. Participants were asked to respond to the statements on a scale ranging from 1 (‘Not at all’) to 5 (‘Very much’). A higher mean score represents higher uncertainty. The Cronbach Alpha scores for this section were 0.76 for the first wave and 0.86 for the third wave.

*The personal initiative (PI)* variable was measured using the tool developed by Frese, et al. [16] and adopted in Hebrew by Hendel, et al. [17]. The original tool comprises two sub-sections: (a) *Self-reported initiative* (seven items) addressing activity and innovativeness in dealing with unexpected difficulties, problem-solving, and achieving goals; and (b) *Passivity* (seven items) representing inactiveness in planning career, adjustment to changing environment, and responding to challenges at work. A panel of experts recommended using only the first part of the tool, while combining two items from the second part that were found relevant to the pandemic. Participants were asked to rank each statement on a 6-point scale ranging from 1 (absolutely disagree) to 6 (totally agree). Items #2 and 8 were recoded. The final score was the mean, with higher scores indicating a higher PI. In an earlier study, the instrument demonstrated good reliability [16, 17]. In this study, Cronbach’s alpha score was 0.73.

*Burnout* was measured using a short 9-item version of the Shirom-Melamed Burnout Measure [18] that was recently used in a national burnout survey [19]. Respondents were asked to rank statements on a scale ranging from 1 (almost never) to 7 (almost always), with higher scores indicating higher burnout. Cronbach's alpha was 0.92.

*Professional functioning* was examined using an 8-item perceived professional functioning scale [14]. The participants were asked to rank the statements on a scale ranging from 1 (strongly disagree) to 5 (strongly agree). Items #3, 5, 6, and 7 were recoded. A high mean score reflects high functioning. The Cronbach alpha scores for this section were 0.76 for the first wave and 0.75 for the third wave.

*Patient Safety and Risk Management policies and practices* under the shadow of the Covid-19 pandemic were measured by a 10-item questionnaire constructed by the authors. The questionnaire comprises a list of 13 actions performed by PSOs in routine work (see “Appendix 1”). Initially, 15 statements were included but five items were removed by the experts' panel due to the differences in PS policies and practices in hospitals and community services. Participants were asked to rank each statement on a 5-point scale ranging from 1 (absolutely disagree) to 5 (totally agree). Items #4, 6, 8, and 10 were recoded. The final score is represented by the mean, with higher scores indicating higher performance of PSO activities. The Cronbach alpha scores for this section were 0.85 for the first wave and 0.81 for the third wave.

*Organizational functioning in Patient Safety and Risk Management* during the Covid-19 pandemic was measured by two items: "The functioning of the organization in the first wave and in the third wave". Participants were asked to rank each statement on a 5-point scale ranging from 1 (very bad) to 5 (excellent). The final score is represented by the mean, with higher scores indicating better organizational functioning with respect to Patient Safety and Risk Management.

*Personal involvement in risk management activities* in the first and the third waves of the pandemic was measured using one question: "To what extent did you deal with risk management and patient safety during the Covid-19 pandemic?" The participants were asked to answer this question using a scale ranging from 1 (not at all) to 5 (to a very large extent), for each wave, separately.

*Qualitative data* was collected by two open-ended questions: "What has helped you to fulfill your PSO role during the Covid-19 pandemic?" and "What are your recommendations for improving the preparation for the next pandemic, with regard to risk management and patient safety?".

### Procedure

The questionnaires were personally distributed by the researcher (DA) and returned manually to the researcher's office. The questionnaires were anonymous. Data collection was at the end of the third wave of the Covid-19 pandemic in Israel, between June-July 2021.

### Ethical approval

Approval for the study was obtained from the Ethics Review Board of the Tel Aviv University, Tel Aviv, Israel (approval no. 0003302-1, 23.05.2021). The approval included an exemption from signing an informed consent form. Initially, the participants were informed that they could terminate their interview at any time and that their interview would be recorded. Partaking in the interview served as a consent to participate in the study.

### Data analysis

Descriptive statistics were used to test the distribution of socio-demographic and study variables. The Kolmogorov–Smirnov test was used to test the normality of the data. The study variables were normally distributed. Differences between groups were measured using a *t*-test for paired samples, a *t*-test for independent samples, and an ANOVA. Pearson correlations (*r*) were used to examine the associations between study variables. Linear regression analyses were used to examine the unique contribution of predictive variables to the explanation of dependent variable variance. A *p* value < 0.05 was considered statistically significant. The data were analyzed using SPSS *v*.28 (IBM, US). STROBE reporting guidelines were used.

*Qualitative analysis*: The qualitative methodology used constant comparative analysis to identify repeated content [20]. A peer debriefing process, in which one of the researchers, who specializes in qualitative research methods (YT), presented the findings to the other researchers, was used to validate the findings. All researchers discussed the findings until they reached a mutual consensus concerning the identified themes and categories, and data saturation was achieved [21].

## Results

### Participants

A total of 84 of the117 PSOs working in Israel healthcare system during the Covid-19 pandemic responded to the invitation to participate in the study, but 6 questionnaires were excluded due incomplete data. Therefore, the convenience sample consisted of 78 PSOs (67%), with an average age of 52.76 years (SD = 10.24, range 31–73), 23.64 (SD = 23.64) years of professional seniority, and 6.24 (SD = 5.34) years of experience in patient safety and risk management. Only 52 (66.7%) of the PSOs were employed full-time in this position. Additional socio-demographic and job characteristics are presented in Table 1.

**Table 1:** Socio-demographic and clinical characteristics of the study population (n = 78)

Variable	Category	N	Valid %
Gender	Male	17	21.8
	Female	61	78.2
Profession	MD	52	67.5
	RN	15	19.5
	Other	12	13.0
	Missing	1	1.3
Workplace	General hospital	28	35.9
	Psychiatric Hospital	12	15.4
	Geriatric center	5	6.4
	Community services	24	30.8
	Other	9	11.5
Percent employment as a PSO	100%	52	66.7
	66–80%	6	7.7
	50%	13	16.7
	25%	5	6.4
	Missing	2	2.6

### Descriptive statistics

Only 51.3% (40) and 57.7% (45) PSOs reported active involvement (4–5 ranking) in PS activities and practice in the first and third pandemic waves respectively. Accordingly, qualitative analysis (reported below) indicated, that other PSOs reduced their involvement in risk management processes or even left their position temporarily in order to return to their primary specialization as clinicians and provide clinical care for Covid-19 patients.

The distribution of the study variables and the differences between the first and third waves of the pandemic are presented in Table 2. PSOs reported high personal initiative and lower levels of burnout (below the score of 3.0 that defines high burnout) [19] than those reported by other healthcare workers (M = 3.4). There were significant differences between the two waves for all the study variables: personal involvement increased, uncertainty decreased, professional functioning improved, as did risk management (RM) and patient safety (PS), practice in the field, and organizational PS function (Table 2). There were no significant differences by gender or profession.

**Table 2:** Study variable distribution and differences between the 1st and 3rd waves (n = 78)

Study variables	Min	Max	M	SD	*t*
Uncertainty, 1st wave	1.86	4.86	3.63	0.70	10.01**
Uncertainty, 3rd wave	1.00	4.43	2.54	0.75	
Professional functioning, 1st wave	1.38	4.50	3.02	0.79	5.71**
Professional functioning, 3rd wave	1.63	5.00	3.47	0.68	
Personal involvement in RM activity, 1st wave	1.00	5.00	3.25	1.41	2.72*
Personal involvement in RM activity, 3rd wave	1.00	5.00	3.69	1.18	
RM and PS practice, 1st wave	1.50	5.00	3.29	0.79	7.68**
RM and PS practice, 3rd wave	1.50	5.00	3.74	0.67	
Organizational PS functioning, 1st wave	1.00	5.00	3.00	0.98	5.13**
Organizational PS functioning, 3rd wave	1.00	5.00	3.63	0.81	
Personal Initiative	3.00	5.00	4.08	0.43	–
Burnout	1.00	5.78	2.93	1.09	–

*p < .01, ** *p* < .001

### Correlational analysis

Correlational analysis revealed a number of interesting findings (Table 3). Uncertainty in the third wave was negatively correlated with professional functioning, RM practice, and organizational PS functioning, and positively correlated with burnout. Burnout was negatively correlated with professional functioning, and RM and PS practice. Personal initiative (PI) was positively correlated with professional functioning in the third wave, as well as with RM and PS practices in both the first and third waves of the pandemic. Organizational PS functioning was positively correlated with RM and PS practice in both waves. There were no significant correlations between the study variables and age, professional seniority, or years of experience in PS and RM (not shown).

**Table 3:** Pearson correlation of the relationships between the study variables (n = 78)

Variables	1	2	3	4	5	6	7	8	9	10	11
1. Personal involvement in RM activity, 1st wave	–										
2. Personal involvement in RM activity, 3rd wave	0.47**	–									
3. Uncertainty, 1st wave	− 0.02	0.17	–								
4. Uncertainty, 3rd wave	− 0.19	− 0.06	0.12	–							
5. Professional functioning, 1st wave	0.14	0.07	− 0.27*	− 0.15	–						
6. Professional functioning, 3rd wave	0.23	0.09	− 0.02	− 0.47**	0.60**	–					
7. RM and PS practice, 1st wave	0.35**	− 0.10	0.01	− 0.39**	0.11	0.27*	–				
8. RM and PS practice, 3rd wave	0.21	− 0.03	0.20	− 0.47**	− 0.03	0.29*	0.78**	–			
9. Personal Initiative	0.11	0.02	0.12	− 0.15	0.15	0.35**	0.31*	0.31*	–		
10. Burnout	− 0.22	0.01	0.11	0.37**	− 0.50**	− 0.74**	− 0.26*	− 0.24	− 0.21	–	
11. Organizational PS functioning, 1st wave	0.35**	0.04	− 0.15	− 0.20	0.10	0.24	0.64**	0.40**	0.16	− 0.25	–
12. Organizational PS functioning, 3rd wave	0.19	0.15	0.13	− 0.32*	0.11	0.36**	0.49**	0.57**	0.17	− 0.20	0.45**

*p < 0.05, ** *p* < 0.01

### Regression analysis

Regression analysis was used to examine the predictors of PS policies and practices in the clinical field as reported by the PSOs. The independent study variables and age were entered into regression separately for the first and the third waves. The results presented in Table 4 indicate that organizational functioning with regard to PS in the first wave, predicted the dependent variable, and explains 43% of the variance. However, during the third wave, the uncertainty variable joined the organizational functioning variable, with the model explaining 37% of the variance of the dependent variable.

**Table 4:** Regression analysis, dependent variable: RM and PS policies and practice, first and third waves

Variables	First wave					Third wave				
	B	St. Err	Beta	*t*	*p*	B	St. Err	Beta	*t*	*p*
(Constant)	0.36	1.36		0.27	0.79	4.25	1.71		2.49	0.02
Age	0.01	0.01	0.05	0.39	0.70	− 0.02	0.01	− 0.23	1.60	0.12
Personal Initiative	0.160	0.219	0.088	0.73	0.47	0.33	0.21	0.20	1.59	0.12
Burnout	− 0.06	0.09	− 0.09	− 0.67	0.51	− 0.12	0.12	− 0.20	1.00	0.32
Uncertainty	0.137	0.144	0.120	0.95	0.35	− 0.41	0.13	− 0.49	3.09	0.004
Personal involvement in RM activity	0.04	0.07	0.08	0.61	0.55	− 0.05	0.07	− 0.08	0.68	0.50
Organizational PS functioning	0.49	0.10	0.64	4.95	< 0.001	0.37	0.12	.42	3.17	0.003
Professional functioning	0.02	0.13	0.02	0.15	0.88	− 0.24	0.23	− 0.25	1.08	0.29

Model for the first wave: R = 0.718, R^2^ = 0.515, Adjusted R^2^ = 0.428

Model for the third wave: R = 0.688, R^2^ = 0.473, Adjusted R^2^ = 0.371

### Qualitative findings

The responses to the two open questions were subjected to a qualitative analysis. The 79 responses received to the first question (see Table 5). were classified according to three main organizational levels: policy and senior management level (32 answers), middle management level (23 replies), and individual level (24 answers). The results presented in Table 5 indicate that three main factors helped PSOs to function during the pandemic: managerial support and cooperation, the mobilization of their team, and the belief in the importance of the position. These three factors were also apparent when the answers were analyzed according to the type of organization: general hospital, ambulatory healthcare services, and psychiatric/geriatric institutions.

**Table 5:** Qualitative analysis of the responses (79) to the open-ended question "What has helped you to fulfill your PSO role during the Covid-19 pandemic?"

Responses		N
Policy and senior management level	Managerial support and cooperation	21
	MOH directives	8
	The ability of the organization to derive lessons learned	2
	The mobilization of the entire organization	1
Middle management level	The mobilization of my team	11
	Cooperation with colleagues	5
	Cooperation with other units	3
	Team-work	2
	Adverse event reports and sharing information	2
Individual level	The belief in the importance of the position	8
	I didn't function as a risk manager	3
	Involvement in the activities	2
	Experience	2
	Self-management: time, tasks, routines	5
	Stress management	2
	No one helped me	1
	Publications and information from abroad	1

A total of 69 recommendations were received in response to the second open-ended question (see Table 6). Notably, the suggestions for improvement focus on reducing gaps that were evident during the first stages of the pandemic: ensure the presence of clear directives from the regulator; establish a professional network of PSOs to share information and considerations; promote active participation in managing the crisis; initiate proactive protocols; and train PSOs to act efficiently in crisis situations. As for the first open question, these points remained relevant when we analyzed the recommendations according to the type of organization: general hospital, ambulatory healthcare services, and psychiatric/geriatric institutions.

**Table 6:** Responses (69) to the question: "What are your recommendations for improving the preparation for the next pandemic, with regard to risk management and patient safety?"

Responses	N
Clear directives from the MOH and distribution of specific information for patient safety officers	14
Cooperation with colleagues and other institutions	10
To attach the patient safety officers team to the pandemic management team	9
To define clearly the duties of patient safety officers in crisis situations	8
To be attentive to the "field" and conduct proactive activities	7
Proper training for patient safety officers as to how to function in crisis situations	6
More patient safety officers positions	4
Acting according to the changing situation	3
Preparation in advance for crisis situations	3
Working according to routines	2
Preparing a dedicated team to cope with the pandemic	1
Planning and prioritizing the activities	2

## Discussion

Healthcare quality and safety experts from Baltimore query the role of quality of care and patient’s safety during the pandemic [22]. Due to the difficulty of obtaining clinical data during a pandemic, it is challenging to assess the quality of the relevant data. However, without systemic learning, it is not possible to improve the processes and results and reach standardization both for the current epidemic and for similar events in the future. In March 2020, the accreditation bodies in the USA announced that they would not monitor the quality and safety indicators in order to allow the hospitals to invest all their means and efforts in the treatment of Covid-19 patients [23, 24]. This move would not have been necessary if the measurement of quality and safety had been digital and had prospectively accompanied the activity itself. For example, quality and safety measurements should have accompanied the orders to administer medicine. The current method of evaluation is retrospective and has two main weaknesses, namely that even if predominantly computerized, it still requires targeted reporting efforts and validation. Feedback to the service providers is provided only after a considerable delay, or even after event completion [25]. During a pandemic, these weaknesses are strengthened in the absence of immediate and continuous systemic learning. For example, there were no answers to the questions of whether steroids and anticoagulants should be used in Covid-19 patients, how remote learning and treatment (Telehealth) could affect the quality of diagnosis and treatment, or whether pronation could prevent intubation and invasive ventilation. In Israel, accreditation surveys by the Joint Commission International (JCI) were postponed, although the national program for quality indicators continued as usual [26].

The findings of this study provide some answers to the questions of the nature of the role of quality, safety, and PSOs during the Covid-19 pandemic and information about whether they were partners in managing the crisis or were sidelined due to changing priorities. It is important to note that the results of the study represent about 500 person-years of experience in the patient safety of PSOs in Israel, if we summarize the relevant results of all the study participants. Nearly half the respondents expressed the opinion that their skills and experience were not fully utilized during the Covid-19 pandemic. They attributed this failing to the preoccupation of the health care system with survival at the expense of patient safety and risk management. Many PSOs moved to work in the Covid-19 departments where they used clinical skills from previous hospital positions, thereby trading their risk management expertise for already forgotten clinical abilities. Thus, many of the PSOs did not take part in the decision-making processes or participate in the management of the medical institution.

The urgent rapid mobilization of the health system to treat the Covid-19 epidemic did not allow sufficient time to coordinate the quality and safety of care required to fight the virus. In addition, the rapid and aggressive changes that occurred generated uncertainty and inefficiency, which in many cases did not contribute to successful treatment. As physicians and nurses with a clinical background, the managers of the quality and safety units were recruited to assist the teams treating patients. Other members of the units, without a similar background, were unable to continue their preplanned annual tasks and instead sought alternative ways to join the battle. This diverted the expertise of quality and safety personnel for example, in the assimilation of a new system or a complicated procedure and global understanding of the health system, which could have been better employed in implementing the urgently needed changes demanded by the pandemic. Quality assessments and, maintaining the patient safety and risk management should be an integral part of the campaign during an epidemic as they are during routine stability. In fact, due to the rapid modifications of protocol required by a pandemic, the system may require continuous improvements in treatment and even more systemic learning.

The absence of clear definitions for PSOs' functions during a medical crisis is something we have seen during the COVID-19 pandemic and was supported by this study. This fact, we assume, explains why many PSOs did not fill their roles during the pandemic and why others had to adjust to local regulations and circumstances in order to support their organization's pandemic response efforts. Therefore, taking into account their unique experience in risk management, we advise that the roles of risk management and patient safety units during major medical crises be defined clearly.

Following the current epidemic, there is room for re-evaluations and the development of clear procedures addressing these issues. In this context, quality and safety experts from France, Australia, and England have proposed a five-step strategy by which the cooperation of quality and patient safety units can contribute to the well-being of patients, staff, and the medical institution [27]:

1. Assessment of readiness and preparation of check lists, collection of data and facts, the establishment of courses and practice, and promotion of staff safety and support.
2. Arrange meetings with patients, members of the patient’s families, and members of the medical team in order to consolidate procedures of protection, social distancing, family visits, and prevention of infection.
3. Improve the quality of care by planning the flow of patients, workshops for caregivers on self-defense issues and treatment, and open decision-making support mechanisms.
4. Reduce the risks to patients through proactive activity, updating guidelines for preventing infections, investigations, and learning from them.
5. Introduce rapid and continuous adjustment of learning means, leveraging options for good patient care, and protecting caregivers against infection, exhaustion, and burnout.

The units for patient safety and risk management have extensive experience in investigating adverse events and in proactive identification of risks in routine medical practice. In emergencies, such "regular" routine adverse events are accompanied by unique adverse events, which may be unexpected and arise from the emergency itself. The work of the patient safety units can be divided into reactive (or retrospective), interactive, and proactive activities, all of which are greatly needed during a crisis. It could be said that the PSO is the manager's adviser on how to investigate, manage, and adapt in real time during the crisis. Responding to issues with quality and safety, such as widespread outbreaks due to the non-ideal emergency facilities and the lack of proper monitoring due to untrained staff, are among the responsibilities of PSOs in an emergency.

In the introduction of this paper, we mentioned the aviation's experience with failure prevention and management. The aviation serves as a model for medical safety and is good for ensuring routine operations of making flying safe and learning from occasional crashes and near misses. Furthermore, there are several crisis management strategies outside aviation that may be taken into consideration. Safety officers for the fire departments, militaries and other first responders typically deal with serious, usually uncontrollable circumstances and must act fast to ensure the safety of the responders, the victims, and to prevent harm to other people. Definitions and procedures of the fire department's health and safety officer, [28] for instance, may serve as a source and an outline for the healthcare systems. This might be an additional model for the rapid response needed during pandemics and other types of crises.

Interestingly, the PSOs in our study reported lower levels of burnout than other healthcare workers. This may be attributed to three main factors that helped them function during the pandemic: managerial support, mobilization of their team, and a belief in the importance of their position. Most of the participants recommended establishing a clear description of the role of a PSO in an emergency, and establishing a professional network of PSOs to share information in real time.

The comparison between performance in the first and third waves reflects the re-organization and adaptation of the health systems to a new threat. As the pandemic progressed, the levels of uncertainty decreased, while personal involvement and professional functioning increased when patient safety practice improved on both a personal and organizational level. Since we did not find research reports on patient safety practice during a pandemic, it was not possible to compare our study findings to the literature. The findings of this study on the links between uncertainty and performance indicate the necessity of enhancing certainty during the Covid-19 pandemic by updating and providing continuing information to PSOs. The situation could have occurred as a result of ambiguity regarding the role of a PSO in a continuing crisis. We assume that the situation could be better controlled and managed in a more efficient and appropriate way by formulating policies and defining the roles of PSOs in advance of a future emergency.

Finally, considering the findings of the study and our opinion, in order to improve the performance of PSOs in emergency, we recommend the following:

- To develop policies and define the role of PSOs in hospitals and the community health services in emergency
- To promote awareness of and implementation of the practice of PSOs' involvement in emergency management and decision-making among senior managers in healthcare
- To develop study programs and to train PSOs to function in an emergency.

## Limitations

This study concerned the perceptions and functioning of PSOs during the Covid-19 pandemic in Israel. It is possible that our results may not fully reflect global practices due to changes in the job description, the background of PSOs, and their positions in their institutes in different countries. Routine quality and safety issues, such as the spread of antibiotic-resistant pathogens or improper patient monitoring that may occur both during pandemics and during regular times, were not included in our survey. In addition, a pandemic is not representative of all crises and may differ significantly from an earthquake or war.

## Conclusions

A crisis inevitably generates uncertainty, a multitude of frequent and urgent tasks, high physical and mental stress due to these many tasks and a lack of knowledge, and the need to adapt policy to changing conditions and the increase in the range and level of risks. We recommend that the risk manager should be physically placed in the "War Room" and receive current information about the situation. He must participate in every important discussion and all structural or functional planning in order to represent the issue of the safety of the staff and the patient and ensure that these issues are addressed from the start. The risk management unit must also conduct daily meetings and patrols in order to monitor/inspect activities and systematically learn from mistakes while optimizing cooperation with relevant units (e.g. the infection prevention unit, medical infirmary, and the pharmacy). There is a need to implement, a clear and well-defined operating protocol for times of crisis and risk management plans for every possible scenario should be prepared in advance. Consideration should be given to updating the roles of the patient safety and risk management units in the community and in the hospitals with a precise definition of their role during a crisis and in different types of crises.

## Data Availability

The data that support the findings of this study are available from the corresponding author [IK], upon reasonable request.

## Appendix 1

See Table 7.

**Table 7:** PS policies and practice during the first and third waves of the Covid-19 pandemic

Items	First wave		Third wave	
	M	SD	M	SD
1. There were training and learning activities addressing patient safety and risk management	3.05	1.37	3.65	1.15
2. The procedures were adjusted for the period of the pandemic	3.82	1.09	3.94	1.01
3. Management conveyed a clear message of the importance and promotion of patient safety	3.42	1.34	3.78	1.24
4. There was ambiguity and confusion about dealing with failures and errors	3.03	1.22	3.94	0.91
5. Reports of potential risks were taken seriously by senior managers	3.71	1.06	4.09	0.89
6. My organization was "forgiving" about the care safety failures	3.40	1.16	3.77	1.19
7. Management-led processes to improve patient safety	3.43	1.13	3.79	0.92
8. Managers did not know how to act in cases of medical errors	3.36	1.20	3.78	1.15
9. I felt that all employees are mobilized to prevent errors and failures	3.07	1.17	3.48	0.95
10. Management policy regarding risk management during the pandemic was not clear	2.95	1.30	3.58	1.31

Higher ranking = better state of policy and practice

## Declarations

### Reporting guidelines

STROBE reporting guidelines were used.

## References

[1] Haklai Z, Goldberger NF, Gordon ES (2022). Mortality during the first four waves of COVID-19 pandemic in Israel: March 2020–October 2021. Isr J Health Policy Res.

[2] Smith CM (2005). Origin and uses of primum non nocere—above all, do no harm!. J Clin Pharmacol.

[3] Saint S, Krein SL, Manojlovich M, Kowalski CP, Zawol D, Shojania KG (2011). Introducing the patient safety professional: why, what, who, how, and where?. J Patient Saf.

[4] Van de Ruit C, Bosk CL (2021). Surgical patient safety officers in the United States: negotiating contradictions between compliance and workplace transformation. Work Occup.

[5] Ministry of Health (MOH). Circular #35/2012: The hospital Patient Safety unit, structure and roles. Ministry of Health, Israel, 2012 (In Hebrew).

[6] Ministry of Health (MOH). Circular #48/2013: The department for treatment safety and risk management at the HMO, structure and roles. Ministry of Health, Israel, 2013 (In Hebrew).

[7] Centers for Disease Control and Prevention (CDC). Underlying medical conditions associated with higher risk for severe COVID-19: information for healthcare professionals. Centers for Disease Control and Prevention (CDC): Atlanta, GA, USA, Feb. 9, 2023. Available at: https://www.cdc.gov/coronavirus/2019-ncov/hcp/clinical-care/underlyingconditions.html. Assessed at March 03, 2023.

[8] Hölscher AH (2021). Patient, surgeon, and health care worker safety during the COVID-19 pandemic. Ann Surg.

[9] Staines A, Amalberti R, Berwick DM, Braithwaite J, Lachman P, Vincent CA (2021). COVID-19: patient safety and quality improvement skills to deploy during the surge. Int J Qual Health Care.

[10] Boserup B, McKenney M, Elkbuli A (2020). The impact of the COVID-19 pandemic on emergency department visits and patient safety in the United States. Am J Emerg Med.

[11] Helmreich RL, Merritt AC. Culture at work in aviation and medicine. National, Organizational and Professional Influences, 2001. Routledge, Taylor & Francis eBooks

[12] GAIN (Global Aviation Information Network) (2000). Operator's Flight Safety Handbook, 2nd issue, GAIN. Available at: https://flightsafety.org/files/OFSH_english.pdf

[13] Kagan I, Kigli-Shemesh R, Bar-Tal Y (2004). Reactions of staff members to the relocation of a psychiatric department into a new building. Psych Serv.

[14] Melnikov S, Shor R, Kigli-Shemesh R, Gun-Usishkin M, Kagan I (2013). Closing an open psychiatric ward: organizational change and its effect on staff uncertainty, self-efficacy, and professional functioning. Perspect Psych Care.

[15] Kagan I, Lancman N, Weisbord I (2022). Experiences and psychosocial predictors of professional function among intensive care nurses under the shadow of Covid-19: a mixed-methods study. J Nurs Scholar.

[16] Frese M, Fay D, Hilburger T, Leng K, Tag A (1997). The concept of personal initiative: operationalization, reliability and validity in two German samples. J Occup Org Psych.

[17] Hendel T, Kigli-Shemesh R, Chor R, Kagan I (2020). Personal and organizational characteristics and their influence on initiative behavior among hospital mental health nurses. Perspect Psych Care.

[18] Shirom A, Melamed S (2006). A comparison of the construct validity of two burnout measures in two groups of professionals. Int J Stress Manag.

[19] Ministry of Health (MOH). The first national burnout survey among healthcare workers. Summary of the findings. Ministry of Health, Israel, 2018. Available at: https://www.health.gov.il/Subjects/HRinHealthSystem/PreventingBurnoutProgram/Documents/102018_a.pdf (In Hebrew).

[20] Glaser BG, Strauss AL (1967). Discovery of grounded theory: Strategies for qualitative research.

[21] Lincoln YS, Guba EG (1985). Naturalistic inquiry.

[22] Austin JM, Kachalia A (2020). The state of health care quality measurement in the era of COVID-19: the importance of doing better. JAMA.

[23] Centers for Medicare & Medicaid Services. COVID-19 and Suspension of Certain Activities Related to the Health Insurance Exchange Quality Rating System, QHP Enrollee Experience Survey (QHP Enrollee Survey) and Quality Improvement Strategy Programs, 2020. Available at: https://www.cms.gov/files/document/covid-qrs-and-marketplace-quality-initiatives-memo-final.pdf

[24] Centers for Medicare & Medicaid Services. CMS announces relief for clinicians, providers, hospitals and facilities participating in quality reporting programs in response to COVID-19, 2021. Available at: https://www.cms.gov/newsroom/press-releases/cms-announces-relief-clinicians-providers-hospitals-and-facilities-participating-quality-reporting

[25] Ministry of Health (MOH). The National Program for Quality Indicators for general and geriatric hospitals, psychiatric hospitals, mother & baby health centers and emergency medical services (ambulances): Annual report for 2013–2021, Ministry of Health, Israel, 2022. Available at: https://www.gov.il/he/departments/publications/reports/quality-national-prog-2013-2021 [In Hebrew].

[26] Konson A, Kuniavsky M, Bronshtein O, Goldschmidt N, Hanhart S, Mahalla H, Peri S, Dollberg S, Niv Y (2022). Quality of care indicator performance was minimally changed in 2020 despite the COVID-19 pandemic. Isr J Health Policy Res.

[27] Carayon P, Perry S (2021). Human factors and ergonomics systems approach to the COVID-19 healthcare crisis. Int J Qual Health Care.

[28] National Fire Protection Association. NFPA 1521: Standard for Fire Department Safety Officer Professional Qualifications, 2020. Available at: https://www.nfpa.org/codes-and-standards/all-codes-and-standards/list-of-codes-and-standards/detail?code=1521

